# Frequency-Domain-Based Structure Losses for CycleGAN-Based Cone-Beam Computed Tomography Translation

**DOI:** 10.3390/s23031089

**Published:** 2023-01-17

**Authors:** Suraj Pai, Ibrahim Hadzic, Chinmay Rao, Ivan Zhovannik, Andre Dekker, Alberto Traverso, Stylianos Asteriadis, Enrique Hortal

**Affiliations:** 1 GROW School for Oncology and Reproduction, Maastricht University Medical Centre+, 6229 HX Maastricht, The Netherlands; 2 Division of Image Processing, Leiden University Medical Center, 2333 ZA Leiden, The Netherlands; 3 Department of Advanced Computing Sciences, Maastricht University, 6229 EN Maastricht, The Netherlands

**Keywords:** medical image translation, unpaired image translation, structure loss, frequency loss, CBCT enhancement, synthetic CT

## Abstract

Research exploring CycleGAN-based synthetic image generation has recently accelerated in the medical community due to its ability to leverage unpaired images effectively. However, a commonly established drawback of the CycleGAN, the introduction of artifacts in generated images, makes it unreliable for medical imaging use cases. In an attempt to address this, we explore the effect of structure losses on the CycleGAN and propose a generalized frequency-based loss that aims at preserving the content in the frequency domain. We apply this loss to the use-case of cone-beam computed tomography (CBCT) translation to computed tomography (CT)-like quality. Synthetic CT (sCT) images generated from our methods are compared against baseline CycleGAN along with other existing structure losses proposed in the literature. Our methods (*MAE: 85.5, MSE: 20433, NMSE: 0.026, PSNR: 30.02, SSIM: 0.935*) quantitatively and qualitatively improve over the baseline CycleGAN (*MAE: 88.8, MSE: 24244, NMSE: 0.03, PSNR: 29.37, SSIM: 0.935*) across all investigated metrics and are more robust than existing methods. Furthermore, no observable artifacts or loss in image quality were observed. Finally, we demonstrated that sCTs generated using our methods have superior performance compared to the original CBCT images on selected downstream tasks.

## 1. Introduction

Radiotherapy is a method of cancer treatment where doses of radiation are targeted at the tumor in order to stop its growth. While delivering radiation to the tumor, healthy organs need to be spared in order to avoid causing damage to them, which makes the design of a radiotherapy treatment a challenging task. Adaptive radiotherapy (ART) is a set of emerging data-driven techniques that aim to improve treatment delivery and outcome by, for instance, administering more accurate treatment plans that account for changes observed during the treatment process. Using onboard imaging data such as cone-beam computed tomography (CBCT) to adapt/re-plan treatment can possibly improve treatment outcomes by better conforming the delivered dose to the tumor and giving a smaller dose to healthy tissues.

However, CBCT images are acquired with about an order of magnitude lower radiation than conventional fan-beam CTs [1] rendering them more susceptible to sources of noise that already affect CT imaging [2]. In addition, the physics of CBCT acquisition introduces sources of noise such as increased scatter because of the use of flat panel detectors. Due to these factors, CBCT images often present lower quality than diagnostic CT scans. Considerable research has been presented on the benefit of processing CBCT images to improve their quality and reduce artifacts with traditional methods such as scatter correction [3], density overrides [4], CT number calibration [4], deformable image registration [5], and certain model-based methods [6]. With the democratization of machine learning (ML) and deep learning (DL), multiple studies have also presented ML/DL-based methods to post-process CBCT images. These methods are often much quicker and less cumbersome than traditional methods and have found wide acceptance in the medical imaging research community. However, in terms of clinical implementation, the vulnerability of ML/DL-based methods in dealing with out-of-distribution data has been a limiting factor. In the section below, a short review of some deep learning approaches along with their benefits and limitations is discussed.

Paired approaches using encoder–decoder networks, specifically UNets [7], have been presented by multiple studies [8,9,10,11]. Kida et al. [8] trained their model with 20 patients in a 2D fashion using a 39-layer UNet architecture and showed that their methods improved the image and dosimetric quality. Image similarity was evaluated through the structural similarity index [12], power-to-signal-noise ratio, ROI mean values, and spatial non-uniformity. Landry et al. [9] compared three UNets trained with different inputs—in projection and image spaces—while using the L2 norm as a loss function in contrast to the MAE. Yuan et al. [10] presented an approach using three cross-sectional slices as three-channel inputs to a two-dimensional UNet. The authors extensively validated their approaches through group-based cross-validation and showed large improvements in image quality across all their test studies. Thummerer et al. [11] trained each 2D network considering different planes—axial, sagittal, and coronal—of the 3D CBCT-CT scan pair followed by aggregation during test time. Although they showed the efficacy of their methods compared to traditional approaches, they did not present comparisons of single plane vs. their multi-planar approach.

Traditionally, the image-to-image translation community has focused on designing handcrafted losses to preserve relevant features in the predicted image. With pix2pix GAN [13], a discriminator was designed to replace complex loss design and preserve high-frequency information. Zhang et al. [14] compared multiple deep learning approaches from UNet encoder–decoders to CycleGANs for CBCT translation and showed that the pix2pix GAN outperforms other methods. Another interesting work by Dahiya et al. [15] showed how physics-based data augmentation can be used to create paired data which can then be leveraged in a pix2pix framework. In addition to improved CBCT, they also generated organ segmentation for the CBCT image. Several studies have incorporated pix2pix GAN to improve the quality of CBCTs and demonstrated their value in clinical downstream tasks [14,16]. A caveat of paired training approaches is the need to obtain paired data in preprocessing, which might pose a hindrance in utilizing all available data efficiently. During treatment, multiple CBCTs are acquired but, generally, only a single pair is a strong candidate when matching with a planning CT. The process of pairing data might also introduce biases such as dependence on the method/quality of registration chosen. These impeding factors along with the emergence of unpaired approaches such as CycleGAN have led the research community to lean toward unpaired approaches.

The CycleGAN framework introduced by [17] is one of the most consistently used unpaired approaches for image-to-image translation. Kurz et al. [18] presented an approach using a 2D CycleGAN where co-registered slices are used as inputs showing strong correspondence with existing CBCT correction methods, both image and dosimetry-wise, while being much faster. Maspero et al. [19] used limited field-of-view CBCTs and rescanned CTs in a purely unpaired fashion across three different anatomical sites. A single network trained on all sites was compared with individually trained networks, both showing a large improvement in image similarity. They additionally showed that the improved CBCT is of sufficient dosimetric quality through dose differences and gamma analysis. Liu et al. [20] demonstrated the use of attention gates in CycleGANs and showed that it improves smoothness and reduces artifacts when compared with a UNet and a vanilla CycleGAN. Various other studies [21,22,23] have also shown the benefit of CycleGAN approaches in CBCT improvement for both visual and dosimetric tasks.

Cycle consistency loss in the CycleGAN is required as infinitely many mappings *G* can produce an output distribution that is identified as belonging to the target domain. Mode collapse is also a common occurrence when solely adversarial mechanics are used for training [17]. Additional structural losses may be added to the CycleGAN, which can further constrain mappings between the source and the target domain. These losses operate directly between the input and its translated image such as (1) the regularization loss proposed by Shrivastava et al. [24], which computes $L_{1}$ distance, and (2) MIND loss [25], which is a dense descriptor-based loss that is crafted specifically for medical image registration. While the $L_{1}$ loss operates in the image domain, the MIND loss operates in the descriptor domain.

In this work, we propose a loss operating in the frequency domain inspired by Jiang et al. [26] and apply it to the use case of CBCT to CT translation. Our main contributions can be summarized as follows:

1. Our proposed frequency structure loss operates in the frequency domain, enforcing constraints where spatial correspondences between images are less sensitive, allowing it to be used effectively on unpaired data.
2. The frequency structure loss improves performance over the baseline CycleGAN and provides images that are more robust than existing methods.
3. The calculation of our loss is faster and less resource-intensive compared to similar losses, such as in Yang et al. [25].
4. Our loss is generalized and does not need any data-dependent configuration, enabling its use for a range of use cases.

We demonstrate the advantage of using our loss through various experiments and show that improved results are obtained in terms of both image-similarity metrics, qualitative analysis, and downstream tasks. The remainder of the paper is organized into the materials and methods (Section 2) where we present methods developed in our work, experiments (Section 3) detailing our configuration and comparative experiments, the results (Section 4) outlining our findings, a discussion (Section 5) of insights obtained from our work, and finally a conclusion (Section 6).

## 2. Materials and Methods

This section describes the model architecture chosen in our work and the implementation details of the proposed loss function. Following this, the methodology used to evaluate our approach and compare its performance with other state-of-the-art approaches is described.

### 2.1. Model Architecture

Generative adversarial networks (GANs) are a category of *generative* models that are trained in an *adversarial* fashion, lending it the name. The generative aspect of a GAN is through a generative network called the generator (*G*) while the adversarial aspect is through a discriminative network called the discriminator (*D*). In the original paper [27], the authors define *G* as a function with parameters $\theta_{g}$ and construct a mapping $G(z;\theta_{g})$ where *z* is a sample from a noise distribution. The discriminator *D* has parameters $\theta_{d}$ and applies the mapping $D(x;\theta_{d})$ to an input *x* providing a scalar output. *x* comes from either the data distribution or the *generated* distribution. The goal of a GAN is to train both *D* and *G* simultaneously such that *D* learns to maximize the probability of identifying whether an input *x* comes from data distribution or the *generated* distribution while G tries to minimize this probability while generating representative samples. This is formally presented as,

(1) $$\begin{array}{cc} L_{adversarial}\left(G,D\right) & =E_{x∼p_{data}}\left[log\left(D\left(x\right)\right)\right]\\ \; & +\;E_{z∼p_{(z}}\left[log\left(1-D\left(G\left(z\right)\right)\right)\right] \end{array}$$

Equation (1) shows the combined objective for the generator and discriminator where $p_{data}$ and $p_{generated}$ are the original (training data) and generated distributions, respectively. The generator tries to minimize this objective while the discriminator tries to maximize it, which is formulated as,

(2) $$\begin{array}{c} G^{*}=\underset{G}{argmin}\;\underset{D}{argmax}\;L_{adversarial}\left(G,D\right) \end{array}$$

#### 2.1.1. Image-to-Image Translation Using GANs

Generative adversarial methods have also been extended for conditional data—where a generated distribution is conditioned on additional information [28]. Several modes of information such as text, labels, and images have been used as conditional information. Pix2pix [13] uses images as input in a conditional fashion to generate translations of those images. Here the sampled noise *z* is conditioned with an input *x*. In addition to the adversarial loss $L_{adversarial}$, an $L_{1}$ loss between the generated and input conditional image is proposed. The combined loss is presented as,

(3) $$\begin{array}{cc} L_{pix2pix}\left(G,D\right) & =L_{adversarial}\left(G,D\right)\\ \; & +\;\lambda E_{x,y,z}[||y-{G\left(x,z\right)||}_{1}] \end{array}$$

The authors interestingly show that the noise *z* does not affect the GAN and can be completely eliminated, leading to providing only *x* sampled from the real data distribution as input. The concept of conditional GANs for image-to-image translation is extended to unpaired settings through the CycleGAN framework.

#### 2.1.2. CycleGAN

The CycleGAN architecture consists of two sets of generator and discriminator networks. Given images belonging to two domains *X* and *Y*, the CycleGAN attempts to learn a mapping from *X*→*Y* through a network *G*. Discriminator $D_{Y}$ learns to differentiate if an image belongs to domain *Y* or not and drives the training of *G*. The concept of cycle consistency is enforced by learning the inverse mapping from *Y*→*X* through a network *F*. Similar to $D_{Y}$, $D_{X}$ exists for the inverse mapping. After mapping *X*→*Y* and *Y*→*X*, the generated image is compared with the original by means of a cycle-consistency loss that ensures accurate reconstruction of the original image through the two mappings.

**Figure 1:**
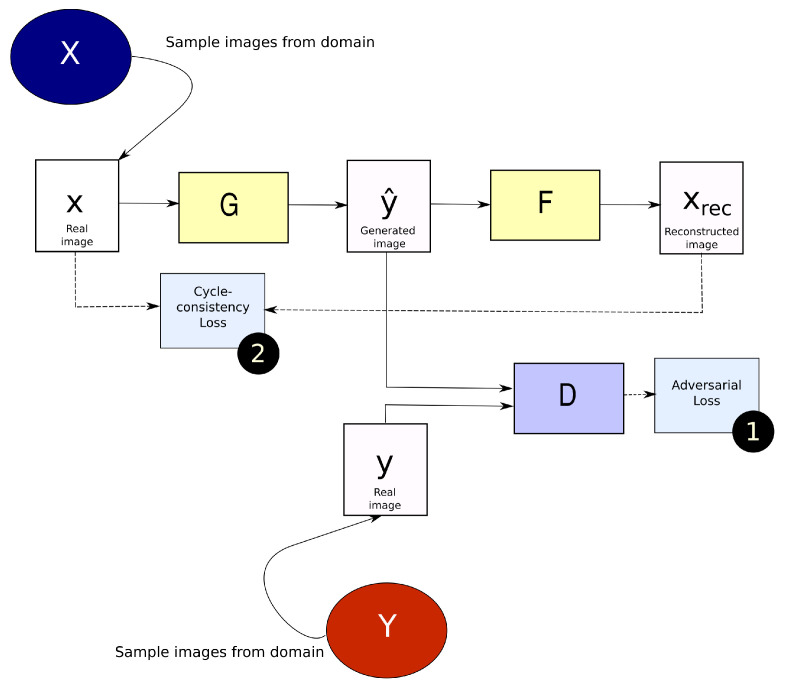
Block diagram of a CycleGAN architecture for the mapping X → Y.

Figure 1 shows a diagram of the CycleGAN architecture for learning a mapping from domain *X*→*Y*. The adversarial loss is similar to Equation (1) but with image *x* from domain *X* as input instead of a noise vector *z*. The cycle-consistency loss in the standard CycleGAN is an $L_{1}$ loss between the input and reconstructed image. In the original paper, the combined loss is formulated as,

(4) $$\begin{array}{cc} L_{CycleGAN}\left(G,F,D_{X},D_{Y}\right) & =L_{adversarial}\left(G,D_{Y}\right)+L_{adversarial}\left(F,D_{X}\right)\\ \; & +\;\lambda_{A}E_{x∼p_{data(x)}}[||F\left(G\left(x\right)\right)-{x||}_{1}]\\ \; & +\;\lambda_{B}E_{y∼p_{data(y)}}[||G\left(F\left(y\right)\right)-{y||}_{1}] \end{array}$$

### 2.2. Generalized Frequency Loss

Frequency spectrum representations of images can allow capturing patterns within the image, that may not be easy to identify in their spatial domain representations. Converting an image to its frequency spectrum representation involves a three-dimensional DFT (discrete Fourier transform),

(5) F(u,v,w)=I(x,y,z);x,y,z∈R

Next, ortho-normalization of the DFT is done as,

(6) $$F^{{}^{\prime }}\left(u,v,w\right)=\frac{1}{\sqrt{LMN}}F\left(u,v,w\right)$$

where *L*, *M*, and *N* are dimensions of the CT and CBCT scans. $F^{{}^{\prime }}\left(u,v,w\right)$ is then shifted such that zero frequency lies at the center of the image. Following the shift, only the magnitude component of the complex frequency spectrum is taken followed by the application of a tanh non-linearity.

(7) $$F_{mag}\left(u,v,w\right)=\left|F^{{}^{\prime }}\left(u,v,w\right)\right|;$$

(8) $$F_{rep}\left(u,v,w\right)=tanh\left(F_{mag}\left(u,v,w\right)\right)$$

The tanh non-linearity is applied in order to scale all values to the range of 0 to 1. This was done to address the differences in the scale of frequency domain representations across different image sets. Alternatively, a careful strategy to ensure that images in the dataset are in similar scales while generating their frequency representations can be designed. However, this becomes extremely data-specific and by no means is generalized. The addition of the tanh makes several assumptions about the importance of different intensities in the magnitude spectrum, as it leads to the following effects: (1) values greater than 0.5 are subdued and (2) as values increase in intensity, their rate of change is also dampened. However, the hypothesis is that the distribution of values, in the magnitude spectrum and not the intensities itself, are of primary importance. Note that the intensity of values is still captured but only higher intensities are dampened. In addition, due to the ortho-normalization of the DFT, the magnitude ranges are reduced. Upon obtaining the generalized frequency representation, the generalized frequency loss is represented as

(9) $$d\left(G\left(x\right),x\right)=\sum_{u,v,w}\left|\right|F_{rep}\left(u,v,w\right)\left(x\right)-F_{rep}\left(u,v,w\right)\left(G\left(x\right)\right){\left|\right|}_{1}$$

where G(x) is the predicted image by the generator, and *x* is the real image. The difference in frequency representations is summed over all voxels in the images. The loss is incorporated into the CycleGAN objective as,

(10) $$\begin{array}{ccc} L_{CycleGAN+freq}\left(G,F,D_{X},D_{Y}\right) & =L_{CycleGAN}\left(G,F,D_{X},D_{Y}\right)\; & +\;\lambda_{f_{A}}E_{x∼p_{data(x)}}\left[d\left(G\left(x\right),x\right)\right]\\ \; & +\;\lambda_{f_{B}}E_{y∼p_{data(y)}}\left[d\left(F\left(y\right),y\right)\right] \end{array}$$

where $\lambda_{f_{A}}$ and $\lambda_{f_{B}}$ are used to balance the contribution of the frequency loss to the overall loss. We set $\lambda_{f_{A}}=\lambda_{f_{B}}=5$ through initial experiments and use it across all configurations unless mentioned otherwise.

### 2.3. Evaluation

Evaluation of unpaired translation methods is a non-trivial task and generally relies on a combination of quantitative and qualitative criteria. Domain-specific evaluation can also often be leveraged in order to determine if the generated images are suitable for downstream tasks.

#### 2.3.1. Image Similarity Metrics

Image similarity metrics common in image translation and quantitative image quality assessment (IQA) [29], namely, mean absolute error (MAE), mean squared error (MSE), normalized mean squared error (NMSE), peak-signal-to-noise ratio (PSNR), and the structural similarity index measure (SSIM) are used to quantitatively evaluate different methods. These metrics are outlined below:

- Mean absolute error (MAE)
  (11) $$MAE\left(ref,pred\right)=\frac{1}{N}\sum_{i=1}^{N}\left|ref\left(i\right)-pred\left(i\right)\right|$$
  where N= total number of voxels in the image. ref, in our work, is the CT image while pred, is the generated image.
- Mean squared error (MSE)
  (12) $$MSE\left(ref,pred\right)=\frac{1}{N}\sum_{i=1}^{N}{\left|ref\left(i\right)-pred\left(i\right)\right|}^{2}$$
  The MSE largely penalizes deviations from the reference image due to the difference being squared.
- Normalized mean squared error (NMSE)
  (13) $$NMSE\left(ref,pred\right)=\sqrt{\frac{\sum_{i=1}^{N}{\left|ref\left(i\right)-pred\left(i\right)\right|}^{2}}{\sum_{i=1}^{N}{\left|ref\left(i\right)\right|}^{2}}}$$
  The NMSE gives the mean squared error while also factoring in the signal power.
- Power-to-signal-noise ratio (PSNR)
  (14) $$\begin{array}{cc} PSNR(ref,pred) & =20\times log_{10}\left(ref_{max}\right)\\ \; & \;-10\times log_{10}MSE\left(ref,pred\right) \end{array}$$
  $ref_{max}$ refers to the maximum value of the ref image. MSE(ref,pred) is computed as described in Equation (12).
- Structural similarity index metric (SSIM)
  SSIM(ref,pred) is computed using the formula presented below, with ref denoted as *x*, and pred as *y*.
  (15) $$SSIM\left(x,y\right)=\frac{\left(2\mu_{x}\mu_{y}+c_{1}\right)\left(2\sigma_{xy}+c_{2}\right)}{\left(\mu_{x}^{2}+\mu_{y}^{2}+c_{1}\right)\left(\sigma_{x}^{2}+\sigma_{y}^{2}+c_{2}\right)}$$
  where μ and σ represent the mean and variance respectively. $c_{1}$ and $c_{2}$ are variables used to stabilize division.

#### 2.3.2. Qualitative Inspection

Qualitative criteria are incorporated into the evaluation procedure, mainly due to the limitations of existing quantitative criteria in capturing *undesired* effects of unpaired translation. Effects such as checkerboard patterns, the addition of artifacts, or the modification of anatomies are not directly captured by metrics. For example, consider a model that translates images that offer good quantitative scores across all metrics. However, this model adds small artifacts such as air pockets that were not present in the original image and cannot be captured by the used metric. As a result, even though the metric score is high, this model will not be accepted clinically. Therefore, qualitative evaluation and analysis are inescapable.

Structured qualitative inspection can allow comparing models in a more systematic manner. Through the analysis of translation from various experiments, a set of criteria for qualitative inspection are formulated:

1. *Presence of artifacts or undesirable elements*: The induction of artifacts is an established drawback of GAN-based generative models [30]. Such artifacts are hard to identify using pixel-based quantitative metrics and, to the best of our knowledge, no other metric that fully captures the range of possible artifacts in a CycleGAN is available. To this end, we inspect images manually to check for artifacts or any undesirable elements such as localized checkerboard artifacts that may appear randomly.
2. *Quality of image in terms of clarity*: This criterion aims to identify the reduction in perceived image quality for translated images. Some very commonly seen phenomena in CycleGAN translations are blurring, aliasing-like effects, and bright spots in parts of the image. Ideally, a reader study would be performed to analyze these factors. However, that is beyond the scope of this study.

Visualizing the entire 3D scan for 18 patients in the test set for multiple trained models would be very time-consuming and impractical. For evaluation purposes, looking at three cross-sectional planes allows us to make a good judgment on the overall quality of the image. Therefore, all the criteria mentioned above are inspected using mid-axial, sagittal, and coronal views.

#### 2.3.3. Out-of-Distribution Analysis

Phantoms are used to test models on out-of-distribution data. Image-similarity metrics are computed on the phantom with an available body mask to generate quantitative metrics. Qualitative inspection of the phantom is done similarly to the patient data, as mentioned in Section 2.3.2. Special attention is paid to the translation of the tumor in the phantom as it is hypothesized to be a potential source of failure for the translation.

#### 2.3.4. Domain-Specific Evaluation

Apart from the quantitative and qualitative evaluations highlighted above, we would like to understand if the proposed methods benefit the use-case of adaptive radiotherapy, for which we chose to design such methods. In order to establish this, we conduct a short analysis of HU value distributions between the original, the target, and the improved CBCT (translated scan). We also compare these scans based on line profiles that demonstrate HU values observed when a line passing through the heart, lung, skeletal muscles, and bones is drawn in the axial plane.

Additionally, the improved CBCT can also be used to generate RT contours through the incorporation of automated segmentation methods. To check if the CBCT improvement benefits this task, we compare the difference in segmentation contours obtained between original and generated images. An automated lung segmentation model [31], trained on a large and diverse dataset, is used to segment the CT/CBCT scan into left and right lungs. Since the test data contains ground truth contours, we generate automated contours for the CT, original CBCT, and improved CBCT and compare each with the ground truth using the Dice score.

## 3. Experiments

In this section, we first outline the datasets used for our work, followed by pre-processing and stratification strategies. We then describe our experimental setup in evaluating the proposed approach. All experiments were run using a configurable YAML-based PyTorch framework called *ganslate* [32].

### 3.1. Datasets

**Figure 2:**
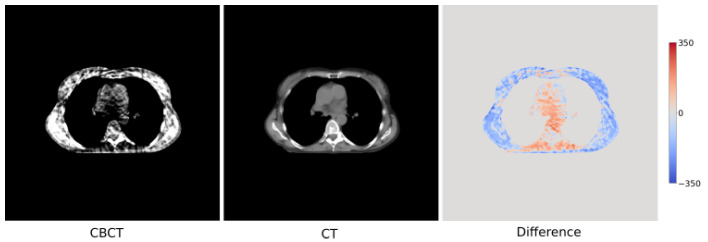
CBCT (**left**) and CT (**middle**) scans for a randomly chosen patient. Differences between the two scans with a HU range of [−300, 300] are also shown (**right**).

A proton beam radiation therapy cohort from MAASTRO Clinic (The Netherlands) comprised of 72 patients diagnosed with lung cancer was selected for our study. At the start of treatment, planning CTs were captured for these patients. During the treatment, CBCT scans were obtained at each fraction resulting in a total of 774 CBCT scans across all patients. In addition to planning CTs, rescanned CTs were also collected to verify/adapt treatment plans, leading to a total of 257 CT scans. Figure 2 shows the CBCT and CT scan from a randomly selected patient. The CBCT images were acquired through Mevion CBCT scanners, which were susceptible to a significant amount of noise while reconstructing the images. These images are shown using a soft-tissue window that helps focus on values within the heart, skeletal muscle, etc. Compared to the CT, the CBCT image has very different intensity values, in terms of Hounsfield unit (HU) calibration along with streaks in the image (due to scattering and motion artifacts). This difference is also highlighted in Figure 2 (right).

**Figure 3:**
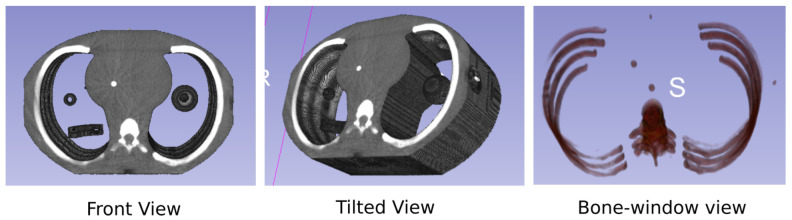
Anthropomorphic phantom shown with front (**left**) and tilted (**middle**) views. A bone-window view is also shown (**right**) where intensities are windowed to expose only the bones. As can be seen, the phantom replicates human-like anatomy.

In addition to the patient cohort, we validate our results on an imaging phantom which represents out-of-distribution data. Imaging phantoms are objects designed with a known geometrical and physical composition which are used for quality assurance and evaluation of CT machines [33]. Due to their known composition, phantoms can be used while eliminating differences induced due to motion, set up, and biological changes. Figure 3 shows the single anthropomorphic phantom that we use for validation.

### 3.2. Data Pre-Processing

The CT scans in the dataset are acquired with different field-of-view settings at the discretion of the radiation therapist resulting in different image spacing grids. This generally needs to be handled to prevent misleading image representations and is done through resampling the image grid. In order to minimize interpolation, the resampling is done as follows,

1. Obtain frequency counts of spacing of all scans in the dataset.
2. Sort spacings in ascending order and rank all spacings based on their frequency counts.
3. Select the smallest rank starting from the bottom of the list.

On following the procedure above, 1.2695 × 1.2695 × 3 (x × y × z) is determined as the ideal spacing. The CBCT scans are also resampled to the resolution of the CT as we expect the CBCT to be a replacement for the CT and hence, possess a similar resolution. All of the above are performed using the *SimpleITK* library [34] in python.

#### Data Stratification for Modeling

The dataset is stratified at the patient level with 50 patients in the training set, 4 in the validation set, and 18 in the test set. Note that due to the availability of more than a single pair per patient, the instances available for training and validation are larger than the number of patients. These are specifically mentioned in the pre-processing subsections.

In order to leverage the full benefits of unpaired data, training is done by selecting a random CBCT and CT scan from the available set of scans and extracting 3D patches of size 16×320×320. Due to this selection strategy, a CT scan from one patient can be paired with a CBCT scan from the same patient or another patient. We hypothesize that this allows for learning more generalized properties and also allows balancing training instances in cases where a particular patient may have fewer CT or CBCT scans. A total of 736 CBCT scans and 219 CT scans from the 50 patients are used during training. A set of *online* pre-processing steps is followed during training starting with masking voxels outside the patient body followed by truncating the field-of-view of the CT image to match the field-of-view of the CBCT image. Finally, we extract patches from both the CBCT and CT images.

For each of the patients in the validation and test set, multiple CBCTs and rescanned CTs are available that are acquired through the treatment process. Rescanned CTs acquired at a time point close to the CBCT generally have good anatomical correspondences although there may be differences due to setup and random errors. These correspondences can be leveraged in order to form weak pairs, which can then be used to evaluate translation quantitatively. The process followed to generate these weak pairs is highlighted below,

1. Select the rescanned CT and the CBCT with the smallest time differences between them (delta). The maximum time difference between the two is limited to one day so that scans with potentially larger anatomical changes are ignored.
2. The rescanned CT is registered to the CBCT through deformable registration using parameters from the *SimpleElastix* library. Parameter files are available at https://github.com/Maastro-CDS-Imaging-Group/clinical-evaluation/tree/master/configs, accessed on 13 May 2021 [35].
3. Apply the registration transform to the rescanned CT and available contours (only available on the test set).

### 3.3. Network Configuration

**Figure A1:**
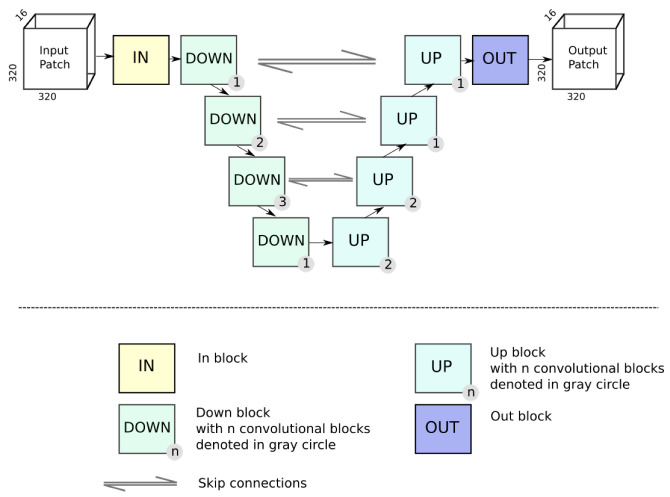
Block diagram of the 3D base configuration architecture. It comprises input blocks, output blocks, up blocks, and down blocks along with skip connections as described in Section 3.3. The input to the network is a patch of size 320 × 320 × 16 and the output is of the same dimension. The legend to the bottom of the dotted line indicates information about individual model blocks.

The network configuration consists of a 3D VNet [36] (shown in Figure A1) as the generator and a 3D PatchGAN as a discriminator. The 3D VNet structure consists of an input block, four down-sampling blocks, four up-sampling blocks, and one out block. The input block consists of a 3D convolutional block of kernel size five followed by instance norm and PReLU. The four down-sampling blocks consist of one, two, three, and two convolutional blocks, respectively, with varying kernel sizes and strides. The four up-sampling blocks consist of two, two, one, and one convolutional blocks. The output block contains two convolutional blocks, the first followed by an instance norm and PReLU and the second followed by a tanh. This configuration is determined based on initial ablation experiments conducted on other medical imaging data with promising performance. Skip connections, similar to the UNet 2D, are also seen in the 3D VNet. The 3D PatchGAN is a 3D version of the 2D PatchGAN, obtained by replacing the 2D convolutions with 3D convolutions. Table 1 shows an overview of the network configuration.

### 3.4. Experimental Setup

We compared our generalized frequency loss against baseline models and previous work in the form of MIND loss [25]. Several configurations of our generalized frequency loss were also evaluated. The experimental configurations are described below:

1. Baseline CycleGAN: The original CycleGAN implementation [17] without any additional structural constraints added.
2. MIND loss: The MIND loss [25] was added as a structural constraint consistent with the authors’ proposed implementation. However, two changes were introduced in the experiment configuration for the MIND loss. In the original work, authors propose a weight of $\lambda_{f_{A}}=\lambda_{f_{B}}=5$. In our experiments, this is changed to $\lambda_{f_{A}}=\lambda_{f_{B}}=50$ through scale-matching with other losses. Additionally, a patch size of (16,192,192) is used for the MIND loss due to memory restrictions.
3. Generalized frequency loss: Our proposed loss was added as a structural constraint to the CycleGAN as outlined in Section 2.2. Two different distance metrics were tested for generalized frequency loss, shown in Equation (9),
  (a) $L_{1}$ distance between the frequency representations;
  (b) $L_{2}$ distance between the frequency representations.
  Other distance metrics such as $L_{p}$ distances may also offer interesting properties but they are not considered in this study.
4. Combined Loss: A combination of Frequency $L_{1}$ loss and the MIND loss is investigated as well. The losses have $\lambda_{f_{A}},\lambda_{f_{B}}$ values consistent with their individual experiments, and are summed to obtain the combined loss. This is trained with a patch size of (16,320,320).

Thus, a total of five different experimental configurations are analyzed.

## 4. Results

**Figure 4:**
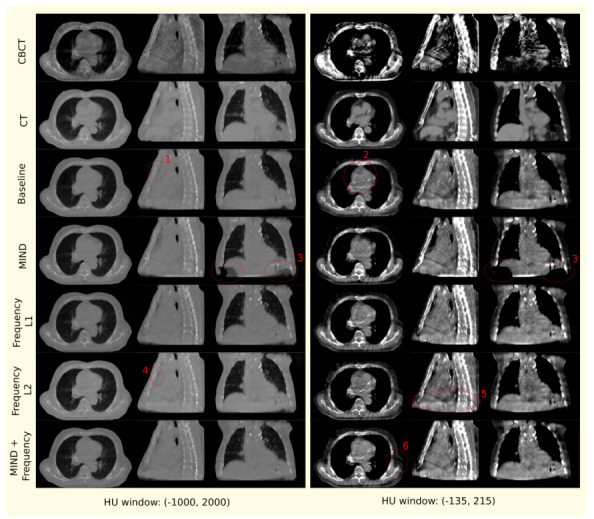
Mid−axial, sagittal, and coronal views for CBCT, CT, and generated images from models with different data-driven constraints for a patient chosen randomly from the test set. Qualitative observations discussed in the text are numbered and marked with red dotted ellipses.

We present the quantitative and qualitative results from the experiments in this section. Table 2 and Figure 4 present the different image similarity metrics and visuals of a patient scan from different experiments.

The lowest deviation from target voxel intensities, in terms of MAE, is provided by the frequency loss $L_{1}$ model. It outperforms the baseline by 4% and improves over other experimental setups on this metric. Squared deviations from the target intensities are measured by MSE, NMSE, and PSNR metrics. The frequency loss $L_{2}$ shows the best performance on these metrics with a 19.7% decrease in MSE and NMSE and a 2.5% increase in PSNR. The second-best performance was shown by the frequency loss $L_{1}$ with a decrease of 18.6% in MSE and NMSE and a 2.2% increase in PSNR. Comparing structural similarity puts the MIND loss model as the strongest one with a 0.009 increase in SSIM over the baseline. The frequency loss $L_{2}$ shows the next best performance with a 0.004 increase in SSIM. The baseline model consistently stands amongst the lowest performed across each of the metrics.

After a visual inspection of the original and generated scans, we make the following observations based on criteria highlighted in Section 2.3.2.

1. Air pockets that are present in the original scan are closed by the baseline model.
2. For the baseline model, a decrease in the quality of the translated image is observed through the addition of checkerboard-like patterns.
3. MIND loss adds unexplained artifacts in the form of black density reduction fields.
4. Frequency $L_{2}$ also closes air pockets similar to the baseline model.
5. Frequency $L_{2}$ provides a shift in density as we move down to the diaphragm, as observed on the sagittal view.
6. MIND + Frequency $L_{1}$ causes a random drop in density across a particular region.

The above observations are made across multiple patients from the test dataset. For the convenience of the reader, only features from a single patient are shown in Figure 4 where several observations can be easily identified. In this figure, the observations are indicated using red dotted ellipses and the numbering scheme utilized in the list above corresponds with the numbers in the figure. It is worth mentioning that although the MIND loss seems to be, visually, the closest to the CT, it adds significant artifacts. The next closest candidate, where no artifacts or image quality drops are observed, is the proposed Frequency $L_{1}$.

### 4.1. Out-of-Distribution Evaluation

**Figure 5:**
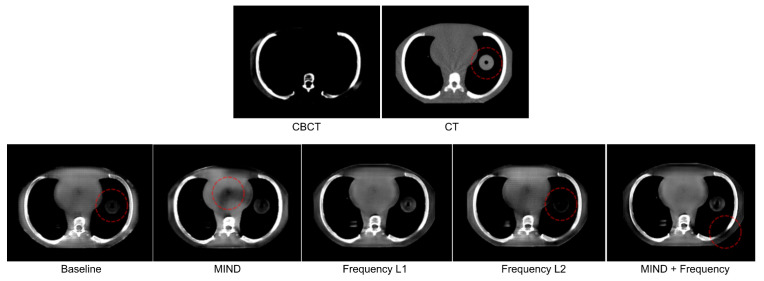
Mid-axial views of the CBCT and CT scans of the phantom shown along with generated images from models with different structure losses. Red dotted circles are used to highlight qualitative observations that are discussed in the text.

Table 3 and Figure 5 show metric scores and visuals of phantoms as outlined in Section 2.3.3.

MIND loss shows the best scores across all metrics. However, when looking at the visuals for the phantom mid-axial slice, MIND loss does not correct values very accurately as can be seen with the black region in the center of the simulated heart. On the other hand, Frequency $L_{2}$ and baseline models do not correct tumor values properly (as indicated by the red circles in the CT, Baseline, and Frequency $L_{2}$ generated samples). Moreover, they also add checkerboard patterns, which can be observed by zooming in on the image. The MIND + Frequency shows regions that are much darker than the CT, as shown by the red circle. Frequency $L_{1}$, similar to observations on the patient data, provides robust translations on the phantom with neither GAN-induced artifacts nor loss of quality. It is interesting to note that the Frequency $L_{1}$ metric values are among the worst across all models.

### 4.2. Domain-Specific Evaluation

In this section, we present the results of domain-specific evaluation criteria as described in Section 2.3.4. The frequency loss with $L_{1}$, which is chosen as the best-performing model, due to its robust performance across both patient and phantom data, is used for subsequent evaluation. The translated scan generated using this model is termed sCT (synthetic CT) and will be used to refer to it henceforth.

**Figure 6:**
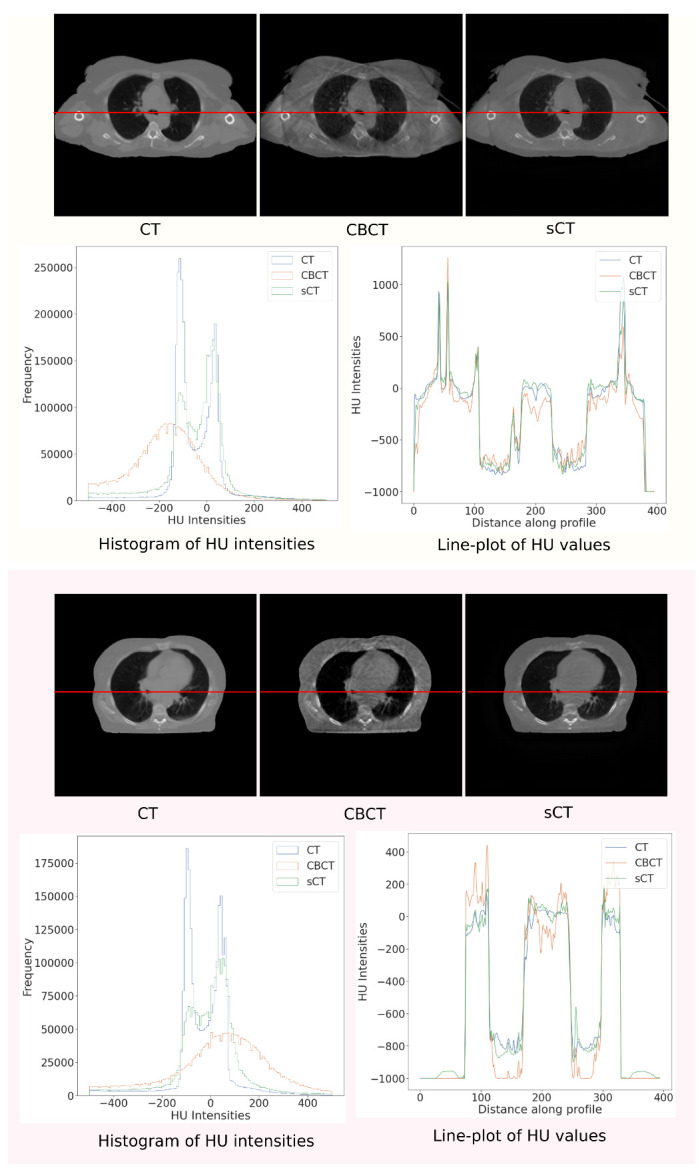
Histogram of HU intensities and line profiles shown for CT, CBCT, and sCT on two patients chosen randomly from the test set. The line chosen for profiling is highlighted by a red line passing the axial view of the images.

**Histogram and line profiles:**Figure 6 shows the histogram of HU intensity values between (−500,500) on the full scan for CT, CBCT, and sCT. In addition, it also shows the line profiles for the same set of scans. The line chosen for the profile is drawn in red over the scans. For both patients, the sCT calibrates well with the CT in terms of the distribution of HU values in the soft-tissue region, made easily observable through the windowing. The sCT also matches the CT line profiles better compared to the CBCT. This behavior extends across all the patients in the test set.

**Figure 7:**
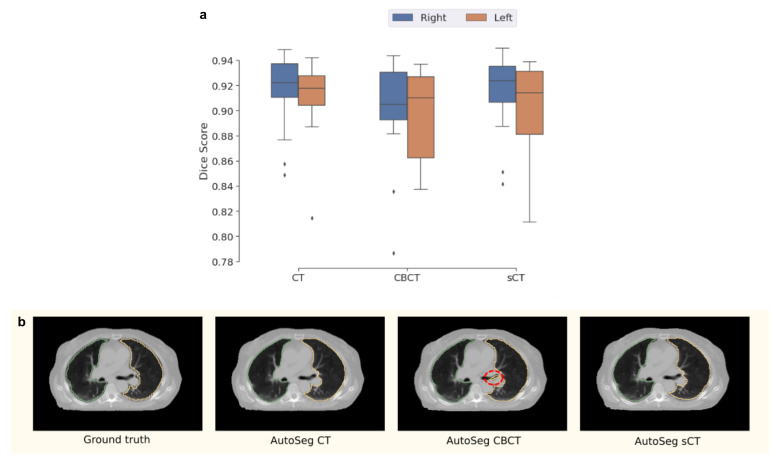
Box plot showing Dice scores for segmentation of left and right lungs using CT, CBCT, and sCT images as input (**a**). Visuals of the segmentation generated by using different inputs to the automated segmentation pipeline along with the ground truth segmentation are also shown (**b**). Note that all segmentations are shown on the CT as the original segmentations were drawn on the CT. The red indication points out a discontuinity in the contour generated on the CBCT image which is rectified when the sCT is used.

**Automated Segmentation:** The sCT is also evaluated on a downstream task of lung segmentation as described in Section 2.3.4. The CT, CBCT, and sCT are contoured for left and right lungs and compared with their ground truth contours, available in the dataset. Figure 7 shows the box plot of Dice scores obtained across all patients along with ground truth and automated contours generated on a randomly selected patient. Table 4 shows the mean Dice scores.

We observed that the sCT, on average, provided improved Dice scores when compared to the CBCT, with an increase of 0.23% and 1.17% on the left and right lung segmentations, respectively (see Table 4). The mean Dice on the sCT even improves slightly over the CT for the right lung. The visualization of segmentation contours in Figure 7 shows a sample case where the CBCT is worse than the sCT (highlighted in red). Note that similar behavior, where CBCT misses/adds parts of the contour, is seen in contours generated across multiple patients in the test set.

## 5. Discussion

In this section, we discuss the results obtained from various experiments conducted in an attempt to provide insights for future studies and applications.

The addition of constraints in the form of structure losses significantly outperformed the baseline CycleGAN as seen through all our experiments. Both quantitative and qualitative results showed improved performance upon the addition of structure losses. This was also seen for out-of-distribution data with the use of phantoms. However, incorporating structure losses with unpaired training data can be challenging as direct image-to-image losses might mislead the training objective. We address this issue by converting image representations to a different domain, namely the frequency domain, where image-to-image spatial correspondences are less sensitive. While we show that the frequency domain loss is a generalized loss as it does not contain any parameters that are data-specific, we only demonstrate its efficacy in the use case of CBCT translation. Follow-up studies investigating its performance across a range of datasets and use cases will allow for a comprehensive determination of its robustness.

We demonstrated that losses built for other medical imaging tasks may not work properly when introduced into the CycleGAN framework. Although MIND loss performed satisfactorily on quantitative scores, it rendered the images unusable due to the large modifications resulting from patient anatomy. In contrast, simple frequency-based losses seem to combine reliably with existing constraints in the CycleGAN and provide translations with desirable qualitative and quantitative scores. Using the $L_{1}$ distance metric while training with the frequency loss provided the lowest mean absolute error on the held-out test set. Similarly, using the $L_{2}$ loss provided the lowest MSE, NMSE, and PSNR metrics, all of which are dependent on squared deviations. This shows that the frequency-based losses translate adequately from their optimization objective on training data to performance on test data. The combination of MIND with frequency-based losses seemed to get rid of the GAN-induced artifacts but the combination performed poorly on quantitative scores and showed other qualitative issues. We note that the MIND implementation we used was different from the authors’ original implementation mainly to allow its balanced contribution of the loss to the overall CycleGAN losses. However, this might be a considerable limitation of this study as we were not able to benchmark against the original implementation.

Another important observation is the insufficiency of solely relying on quantitative analysis in choosing the best model. This is observed even with out-of-distribution data, where strong pairs were formed. For instance, Frequency $L_{1}$ provides one of the worst scores on the phantom but it is superior to the other models as it does not induce artifacts or result in a reduction in quality that all other models were susceptible to. This puts forward the question of whether existing image similarity metrics can be relied on fully to evaluate such methods. Research into evaluation methods that can sufficiently capture these properties in generated images would push the field closer toward general and clinical acceptance. Gragnaniello et al. [37] present a review of existing methods for synthetic image detection and propose potential research areas for the future. These methods could also help in quantitatively determining undesirable additions such as artifacts in the generated images.

Domain-specific methods of evaluation can provide good insight into the clinical usability of a particular set of methods. As seen in Section 4.1, synthetic CT generated from the best-performing model provided HU intensity distribution and line profiles in line with real CTs. Automated segmentation on the synthetic CT showed performance on par with real CT (even better for the right lung) and improvements from the original CBCT. Given the simplicity of the translation process, it can be integrated into existing clinical workflows to improve the quality of the CBCT. The improved CBCT can be useful for multiple downstream tasks, from improving auto-contouring to adapting treatment plans.

## 6. Conclusions

In this study, we investigated structure losses for CycleGAN-based CBCT enhancement comparing several different types of structure losses. We proposed a frequency domain structure loss that is generalized and does not depend on specific datasets for parametrization. The addition of this loss improved MAE, MSE, NMSE, and PSNR, by 4%, 20%, 18%, and 3%, respectively, compared to the baseline. The generalized frequency loss, implemented as a part of this study, proved to not only improve over the baseline but also outperform existing methods, such as the MIND loss [25]. This was done at a much lesser memory and computing cost. More importantly, in terms of qualitative comparison, it provided the best performance, with no drops in image quality or any addition of artifacts.

We also used out-of-distribution data in the form of imaging phantoms to demonstrate the robustness of methods compared in this study. The generalized frequency loss showed the best qualitative performance but fell short in terms of quantitative performance when compared to the MIND loss. One of the core goals of this study was to develop a reliable and robust method for CBCT translation. This improved CBCT can benefit various adaptive radiotherapy workflows in the clinic such as auto-contouring, image registration, and dosimetry. Improvements in these workflows can save clinicians valuable time and effort along with a reduction of costs associated with repeated imagery. Improved CBCT can not only benefit clinics but also patients as improved quality CBCTs can mean fewer CT scans and, therefore, lesser radiation exposure. We implemented clinically motivated evaluations such as HU intensity distribution comparisons and line profiles, where we demonstrated that the improved CBCT matches the fan-beam CT accurately. Furthermore, the value of improved CBCTs in downstream tasks was shown through a comparison of contours generated through lung auto-segmentation. The mean Dice scores of contours on the improved CBCT were comparable to the fan-beam CT and surpassed the CBCT.

We recommend several potential directions for extending our work in the future. First, a full dosimetric evaluation of our methods would better establish the applicability of our methods in the clinic. However, designing clinically acceptable treatment plans can be quite complex, and, therefore, we exclude them from this study and propose it as future work. As we provide our models and code, integrating our pipeline into treatment planning workflows can be relatively straightforward and can drive further investigation in this direction. Second, a broader evaluation of our frequency loss with different data and use cases will allow us to present it as a truly generalized method experimentally. Medical imaging use cases with different modalities such as MRI and PET-CT might benefit largely from the use of such a frequency loss. We provide a simple function to generate frequency loss terms which can then be added to existing losses, thereby allowing research to incorporate and compare its benefit. Finally, further exploration into the development of domain-specific quantitative metrics that can capture artifacts in generated images is needed. In addition to standard image similarity metrics, we rely on qualitative evaluation using domain expertise. However, this can prove to be quite challenging for large-scale datasets, limiting the scope of extensive validation. We suggest further research into developing novel evaluation criteria for CT images that can look at image similarity, and organ- and tissue-specific similarity in an automated manner. For example, an existing deep learning segmentation method can be used to segment several organs of interest, and their values can be analyzed individually and in a collection to determine overall quality.

## Figures and Tables

**Table 1:** Base configurations used for the experiments performed across the two datasets.

Generator	VNet 3D
Discriminator	PatchGAN 3D
Learning rate	D: 0.0002, G: 0.0004
Batch size	1
LR schedule	Fixed for 50%, Linear decay for 50%
Optimizer	Adam $\left(\beta_{1}=0.5,\;\beta_{2}=0.999\right)$
Lambda ($\lambda_{A},\lambda_{B}$)	5
Input size (z, x, y)	(16,320,320)
Normalization	Instance normalization
Training iterations	30,000

**Table 2:** Quantitative metrics obtained on the test set for experiments with various structure losses run on the CBCT-CT dataset. All metrics were computed between images with their intensities expressed on the HU scale and clipped into the range [0, 3000]. The best value per metric is highlighted in bold letters and the second best value per metric is italicized.

Model	MAE	MSE	NMSE	PSNR	SSIM
Baseline	88.85	24,244	0.031	29.37	0.935
MIND	85.91	25,604	0.032	29.27	**0.944**
Frequency loss $L_{1}$	**85.50**	*20,433*	*0.026*	*30.02*	0.935
Frequency loss $L_{2}$	*85.97*	**20,247**	**0.027**	**30.12**	*0.938*
MIND + Frequency loss	86.63	21,125	0.027	29.88	0.935

**Table 3:** Image similarity metrics on the phantom for experiments with various structure losses. All metrics were computed between images with their intensities expressed on the HU scale and clipped into the range [0, 3000]. The best value per metric is highlighted in bold letters.

Model	MAE	MSE	NMSE	PSNR	SSIM
Baseline	72.16	16207	0.024	34.55	0.976
MIND	**62.74**	**11,303**	**0.017**	**36.12**	**0.985**
Frequency loss $L_{1}$	71.39	16,878	0.025	34.38	0.976
Frequency loss $L_{2}$	63.65	12,046	0.018	35.84	0.983
MIND + Frequency loss $L_{1}$	75.34	17,723	0.027	34.16	0.975

**Table 4:** Mean Dice scores for left and right lung segmentations for the CT, CBCT, and sCT images as input to the automated segmentation model.

	Left Lung	Right Lung
CT	**0.910**	0.913
CBCT	0.898	0.902
sCT	0.900	**0.915**

## Data Availability

As the datasets used in this work are private, we do not provide any resources attached to the data. Our results on the test set can be viewed as a Weights and Biases report at https://api.wandb.ai/report/surajpai/w0rojf7d.
